# 321. Clinical Characteristics of Hospitalized HIV Patients with COVID-19 in Miami

**DOI:** 10.1093/ofid/ofab466.523

**Published:** 2021-12-04

**Authors:** Folusakin Ayoade, Jose G Castro, Priscilla Valls, Tanya R Quiroz, Annette Amoros

**Affiliations:** 1 University of Miami, Miami, Florida; 2 Jackson Health System, Miami, Florida

## Abstract

**Background:**

HIV is a significant risk factor for acquiring SARS-CoV-2 infection and is associated with increased risk of mortality from COVID-19. Information on the clinical characteristics of persons living with HIV(PLWH) hospitalized due to COVID-19 infection are inconsistent and sparse. As Miami area is currently the epicenter of new HIV infection, an understanding of the clinical characteristics of COVID-19 in hospitalized HIV patients in South Florida is needful.

**Methods:**

This is a single center retrospective case series analysis of individuals with HIV hospitalized with COVID-19 from March 1, 2020 to March 31, 2021. We analyzed relevant data related to demographics, comorbidities, clinical presentation, HIV viral load and CD4 profiles, serum inflammatory markers, COVID-19 treatment and survival.

**Results:**

25 patients were identified. The demographic, socioeconomic and clinical data are described in Table 1. 88% of subjects. were on HIV antiretroviral treatment (ART) but only 60% had CD4 counts > 200cells/mm^3^. More study results are shown in Figures 1 and 2. The serum ferritin ranged from 29 to 40,577ng/mL while serum creatinine ranged from 0.51 to 2.8mg/dL, mean 1.04± 0.46 mg/dL. The Pearson correlation between serum ferritin and serum creatinine (SCreat) was 0.715, p < 0.001 and between lymphopenia and SCreat, it was 0.544, p=0.005. 40% of subjects with CD4 < 200 cells/mm^3^ died compared to 33% with CD4 > 200 cells/mm^3^.

Figure 1. Bar chart showing month and year of hospital admission for COVID-19 in HIV infected persons

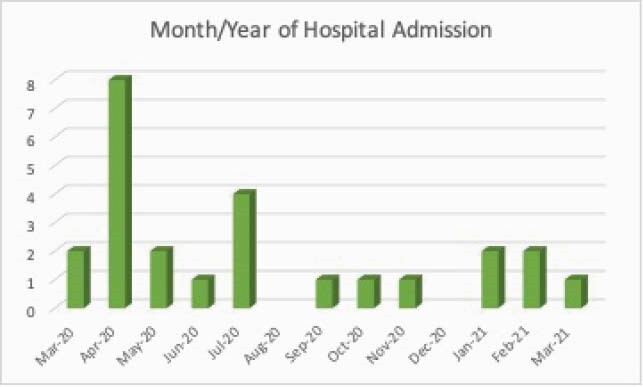

Table 1

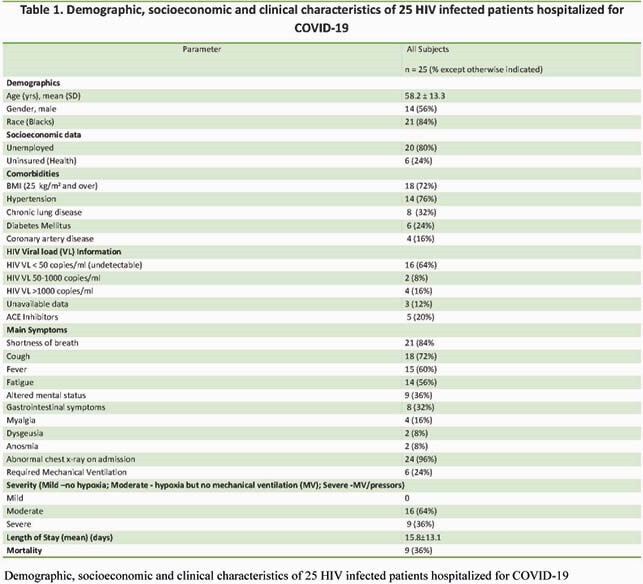

Figure 2. Bar chart showing different percentages of the cohort who received the different COVID-19 treatment illustrated

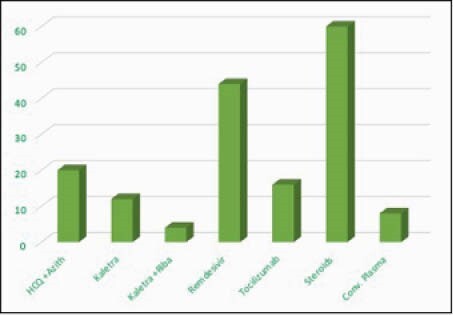

**Conclusion:**

This first case series of hospitalized COVID-19 patients in PLWH illustrate important demographic and socioeconomic trends with an imbalance towards African Americans. The group mortality rate appear to be higher compared to the overall mortality rate of COVID-19 reported in the general population or other published HIV-COVID-19 coinfection case series. This is not surprising given the fact that only 64% of the cohort had undetected viral load and only 60% had CD4 counts > 200 despite reported 88% ART use. Correlations between lymphopenia and serum ferritin on one hand and serum creatinine on the other hand should be further explored in a larger case series or prospective study. Since COVID-19 mortality is related to HIV severity, improving socioeconomic status and ART compliance could play a big role in positively improving outcome of hospitalized HIV-COVID 19 patients.

**Disclosures:**

**All Authors**: No reported disclosures

